# Effects of Gastric Acid and Antiacid Medications on Surface Roughness, Morphology, and Optical Properties of Resin-Based Materials

**DOI:** 10.3390/polym18060756

**Published:** 2026-03-20

**Authors:** Ezgi Tüter Bayraktar, Ayşe Aslı Şenol, Elif Alkan, Bengü Doğu Kaya, Dilek Tağtekin

**Affiliations:** 1 Department of Restorative Dentistry, Faculty of Dentistry, Marmara University, İstanbul 34854, Türkiye; asli.tuncer@marmara.edu.tr (A.A.Ş.); elifalkan111@gmail.com (E.A.); dtagtekin@marmara.edu.tr (D.T.); 2 Department of Restorative Dentistry, Faculty of Dentistry, Çanakkale Onsekiz Mart University, Çanakkale 17100, Türkiye; bengu.dogukaya@comu.edu.tr

**Keywords:** antiacid medicaments, fluorescence, gastric acid, surface morphology, surface roughness, translucency

## Abstract

Effects of gastric acid and antiacid medications on the surface and optical properties of resin-based restorative materials were evaluated. A hybrid-CAD/CAM block, a 3D-printed resin, a paste-type composite, and a flowable composite were investigated (n = 9). Samples were prepared (1 mm thickness) and polished. All samples were exposed to gastric acid for 6 days, followed by a second exposure to distilled water, antiacid medication, or gastric acid for 56 min. Surface roughness, translucency, and fluorescence were assessed at baseline (T_0_), after gastric acid exposure (T_1_), and after the second exposure (T_2_). Surface morphology was examined by scanning electron microscopy. Data were analyzed with a significance level of *p* < 0.05. Gastric acid exposure caused a significant increase in surface roughness and a significant reduction in translucency in all materials (*p* < 0.05). CAD/CAM and paste-type composites exhibited significantly higher roughness values than the 3D-printed and flowable composites (*p* < 0.001). Fluorescence changes were observed in all groups, but the highest ΔE_00_ values were observed in the 3D-printed and flowable composites (*p* < 0.001). Gastric acid adversely affected the surface and optical properties of resin-based restorative materials, while antiacid medication showed limited, material-dependent protective effects.

## 1. Introduction

Gastroesophageal reflux disease (GERD) is a common condition whose prevalence has increased in recent years, influenced by factors such as stress and dietary habits. In patients with GERD, gastric acid may reach the oral cavity, where it exhibits extremely low pH values ranging from 1 to 2. Owing to its highly acidic nature, gastric acid has been shown to affect enamel, dentin, gingival tissues, and restorative materials [1,2,3]. In patients exposed to erosive challenges, the long-term performance of restorative materials is considered clinically important in terms of both esthetic outcomes and functional durability [4]. Although several studies have investigated the effects of gastric acid and acidic solutions on restorative materials in recent years [5,6,7,8,9], the continuously expanding diversity of dental materials indicates that the effects of gastric acid and medications used in the management of GERD on different contemporary restorative materials have not yet been fully clarified. It is well established that exposure to gastric acid, as well as to acidic environments or solutions, can weaken the matrix–filler interface of dental restorative materials, increase surface roughness with a subsequent rise in plaque accumulation, and consequently promote plaque accumulation, and potentially compromise surface hardness and mechanical properties [9,10,11]. In addition, it has been reported that exposure to acidic environments may affect not only the physical and mechanical properties of restorative materials but also their optical characteristics, such as translucency and fluorescence, as a result of changes in surface morphology [10]. Contemporary restorative materials exhibit substantial differences not only in their clinical applications but also in their manufacturing techniques and microstructural characteristics. Accordingly, the optical, mechanical, and surface properties of restorative materials are influenced by both the environments or solutions to which they are exposed and their intrinsic composition and production methods [12,13]. In resin composites commonly used for direct restorations, material properties are influenced by the resin matrix–filler interface, monomer composition, filler content, and viscosity characteristics, including whether the material is formulated as a conventional paste or as a flowable composite [14,15].

Similarly, recent advances in indirect restorative materials have involved the development of both additive and subtractive manufacturing techniques, leading to an increasing diversity of available materials [16]. CAD/CAM blocks used in subtractive manufacturing consist of a ceramic-filled resin matrix and are widely applied in various restoration types, including inlays, onlays, and overlays. The coexistence of these two distinct phases may enhance mechanical properties while also influencing the material’s behavior in acidic environments [17]. The response of these materials to long-term acidic exposure in the oral environment is of clinical relevance for the longevity of restorations [11]. In contrast, additive manufacturing in dentistry has increasingly involved the use of 3D-printed resins, whose mechanical, optical, and surface properties may vary depending on factors such as matrix composition, polymerization processes, and post-curing procedures [18]; consequently, their behavior in acidic environments may also differ [19,20]. However, there is still limited knowledge about how different types of restorative materials such as direct composites, CAD/CAM blocks, and 3D-printed resins respond to exposure to gastric acid in GERD patients as well as to medications used in its management. In particular, only a few studies have investigated the combined effects of gastric acid and alginate-containing antiacid treatments on the surface and optical properties of these materials. In addition, the effect on fluorescence, which is an important factor for the esthetic appearance of restorations, has rarely been examined under these conditions. In light of these considerations, the aim of this in vitro study was to evaluate changes in surface roughness and surface morphology, as well as optical properties (translucency and fluorescence), of resin-based materials with different compositions and manufacturing techniques following exposure to gastric acid and medications used in the management of GERD. The hypotheses (H_1_) of the study were as follows:

(I) Gastric acid exposure affects the surface roughness and morphology of resin-based materials.
(II) Gastric acid and antiacid medications alter the translucency and fluorescence of resin-based materials.
(III) Resin-based materials exhibit material-dependent surface and optical responses to gastric acid and antiacid exposure.

## 2. Materials and Methods

A CAD/CAM block (Grandio Blocs, VOCO GmbH, Cuxhaven, Germany), a 3D-printed resin (TriniQ, BEGO GmbH, Bremen, Germany), a paste-type resin composite (GrandioSO, VOCO GmbH, Germany), and a flowable resin composite (G-aenial Injectable, GC Corp., Tokyo, Japan) were investigated (Table 1). All materials were selected in the A2 shade. The sample size of this study was determined using G*Power version 3.1.9.7. software. The minimum required sample size was calculated as n = 9, with a statistical power of 95% (1 − β), a confidence level of 95% (1 − α), and an effect size of f = 0.74 [21,22].

CAD/CAM block samples were cut into 1 mm thick slices using a diamond saw (Isomet, Buehler Ltd., Lake Bluff, IL, USA) under water cooling. Samples of the resin composite groups were prepared using aluminum molds with standardized dimensions of 1 mm in thickness and 8 mm in diameter. The resin composites were placed into the molds, and the outer surfaces were covered with a Mylar matrix strip and glass slides to obtain flat and uniform surfaces. Polymerization was performed using an LED light-curing unit (Elipar Deepcure-S, 3M ESPE, St. Paul, MN, USA) at a light intensity of 1470 mW/cm^2^ for a duration of 20 s, with the tip in direct contact with the glass surface. The 3D-printed resin samples (1 mm thickness, 8 mm diameter) were fabricated using a Digital Light Processing printer (Max UV, Asiga, Sydney, Australia) with a layer thickness of 50 µm and a build orientation of 0°. Post-processing included immersion in isopropanol in an ultrasonic bath (Foshan Adelson Medical Devices, Foshan, China) for two cycles of 3 min each. The specimens were subsequently polymerized using an Otoflash G171 unit (NK-Optik, Baierbrunn, Germany), with 1500 flashes applied to each side at a frequency of 10 Hz. The polishing procedure was carried out using diamond-impregnated polishing spirals (DiaComp Plus Twist, Eve, Keltern, Germany) for all groups.

After the sample preparation, all groups were stored in distilled water at room temperature for 24 h. Subsequently, all samples were immersed in simulated gastric acid (0.06 M HCl, pH 1.2) at 37 °C for 6 days (144 h) as an extended erosive challenge, following protocols designed to model long-term GERD-related HCl exposure and simulate cumulative GERD-related acidic challenges [23].

After this initial gastric acid challenge, specimens were randomly allocated into three groups according to the second exposure condition (Table 2): distilled water, antiacid medication (Gaviscon; sodium alginate, sodium bicarbonate, calcium carbonate, pH 8), or gastric acid. The second exposure was performed for a total of 56 min [24], corresponding to an immersion-cycle regimen of 2 min/day for 28 days, which has been reported to simulate approximately 2 years of clinical exposure to antiacid medications (assuming an average intraoral contact time of 5 s per use). Throughout the immersion procedures, specimens were maintained at 37 °C in a shaking incubator (ZWYR-240, LABWIT Scientific, Sydney, Australia), and the pH of the solutions was monitored daily to ensure stability.

### 2.1. Evaluation Periods

**Figure 1:**
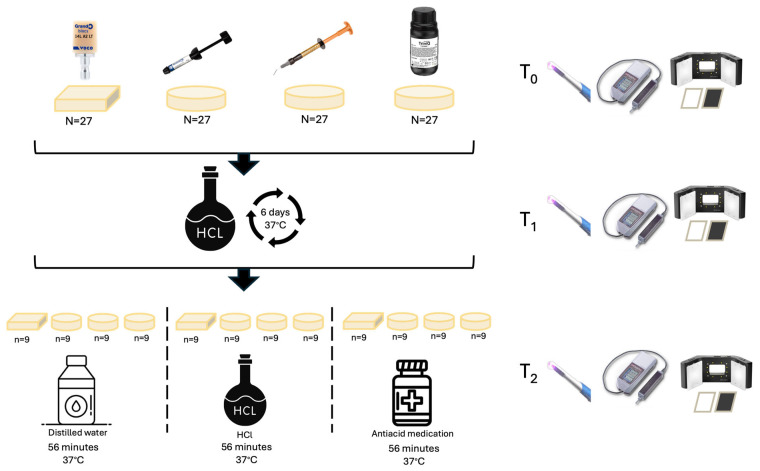
Schematic illustration of the study.

Translucency, fluorescence, and surface roughness measurements were performed for each specimen at three different time points. Baseline measurements (T_0_) were recorded before any procedure. Following gastric acid exposure (T_1_), measurements were repeated. Final measurements (T_2_) were obtained after the second exposure (Figure 1).

### 2.2. Surface Roughness

Surface roughness (Ra) values were measured using a contact profilometer (2-μm-diameter, 90° diamond tip at a speed of 1 mm/s, a force of 0.7 mN, a tracing length of 4 mm and a cut-off length of 0.25 mm) (MarSurf PS10, Mahr, Göttingen, Germany). Three surface roughness measurements, rotating the sample by 60 degrees (in a clockwise direction), were recorded for each specimen, and the mean value was calculated in μm.

### 2.3. Translucency Evaluation

The translucency of the materials was evaluated using the Translucency Parameter (TP), which represents the color difference between measurements obtained over standardized white and black backgrounds. Color measurements were performed using a dental spectrophotometer (VITA Easyshade V, VITA Zahnfabrik, Bad Säckingen, Germany). Measurements were obtained by placing the probe tip perpendicular to the specimen surface to minimize external light interference. CIE L*, a*, and b* values were recorded for each specimen against both backgrounds. The TP_00_ was calculated using the CIEDE2000 color difference formula [25]. The parametric factors k_L_, k_C_, and k_H_ were set to 1, as the measurements were performed under standardized and constant experimental conditions [26].

### 2.4. Fluorescence Evaluation

The fluorescence properties of the samples were evaluated using a Qray Pen device (AIOBIO, Seoul, Republic of Korea). Images were captured in a dark room under fixed camera settings and standardized positioning to ensure consistency and reproducibility at each time point. The acquired images were transferred to image analysis software (Adobe Photoshop, Adobe Systems, San Jose, CA, USA), and the L*, a*, and b* color coordinates were obtained. Color differences between the different time points were calculated using the CIEDE2000 (ΔE_00_) formula. Each measurement was repeated three times per specimen, and the mean values were used to calculate color differences.

### 2.5. SEM Analysis

Representative samples from each group were selected for scanning electron microscopy (SEM) analysis to qualitatively evaluate surface morphology changes following the experimental procedures. Samples were analyzed using a scanning electron microscope at magnifications of ×5000 and ×10,000, with an acceleration voltage of 10 kV. Prior to imaging, all samples were coated with a thin layer of gold to enhance surface conductivity and image quality. The sample surfaces were sputter-coated with a thin gold–palladium (Au/Pd) layer using a coating unit (EM ACE200, Leica Microsystems, Wetzlar, Germany). The coated specimens were then examined under a scanning electron microscope (Zeiss EVO-MA 10, Zeiss, Oberkochen, Germany).

### 2.6. Statistical Analysis

The statistical analysis was performed using IBM SPSS V25. The normality of data distribution was assessed with the Shapiro–Wilk test. Translucency, fluorescence difference and surface roughness (Ra) was analyzed using generalized linear models including material type (Grandio Blocs, GrandioSO, G-ænial Universal Injectable, TriniQ), application type (acid, Gaviscon liquid, distilled water), measurement time (Baseline-T_0_/after acid challenge-T_1_/after application-T_2_), and their interactions. Multiple comparisons were evaluated with Tukey’s post hoc test. The level of significance was set at *p* < 0.05.

## 3. Results

### 3.1. Translucency

The ANOVA model for translucency was statistically significant (*p* < 0.001), explaining 73.6% of the total variance (R^2^ = 0.736). Material type significantly affected translucency values (*p* < 0.001). Application type also showed a statistically significant effect (*p* = 0.010), and measurement time had a highly significant influence (*p* < 0.001). The descripted statistics are presented in Table 3 and detailed ANOVA results are summarized in Table 4. Significant interactions were detected between material and application (*p* < 0.001), material and time (*p* < 0.001), and application and time (*p* = 0.008). However, the three-way interaction among material, application, and time was not statistically significant (*p* = 0.736) (Table 4).

Post hoc Tukey analysis revealed statistically significant differences among all material groups. GrandioSO demonstrated the highest translucency values, followed by TriniQ, G-ænial Universal Injectable, and Grandio Blocs. Regarding the solution groups, translucency values in the Gaviscon liquid group were significantly higher than those in the distilled water group (*p* = 0.012), whereas no statistically significant differences were found between acid and Gaviscon liquid (*p* = 0.059) or between acid and distilled water (*p* = 0.821).

For the time factor, significant differences were observed among all measurement periods. Baseline values were significantly higher than those measured after acid challenge and after applications (*p* < 0.001), and values after acid challenge were significantly higher than those recorded after application (*p* = 0.003).

### 3.2. Fluorescence

The three-way ANOVA revealed that the overall model was statistically significant (*p* < 0.001), explaining 54.2% of the total variance (R^2^ = 0.542). The type of material significantly affected difference in fluorescence values (*p* < 0.001), whereas the type of application had no significant effect (*p* = 0.521). Measurement time significantly influenced fluorescence difference (*p* = 0.002). Detailed descriptive statistics are presented in Table 5 and ANOVA results are shown in Table 6.

A significant interaction was observed between material and application (*p* < 0.001), while the interactions between material and time (*p* = 0.806), application and time (*p* = 0.286), and the three-way interaction among material, application, and time (*p* = 0.910) were not statistically significant (Table 6).

Post hoc Tukey comparisons showed that G-ænial Universal Injectable and TriniQ exhibited significantly higher difference in fluorescence values than Grandio Blocs and GrandioSO (*p* < 0.001). However, no statistically significant difference was detected between Grandio Blocs and GrandioSO (*p* = 0.337) or between G-ænial Universal Injectable and TriniQ (*p* = 0.439).

### 3.3. Surface Roughness

The three-way ANOVA model for surface roughness was statistically significant (*p* < 0.001) and explained 88.7% of the variance (R^2^ = 0.887). Material type showed a strong influence on surface roughness (*p* < 0.001, partial η^2^ = 0.882). Both application type (*p* = 0.025) and measurement time (*p* < 0.001) also had significant effects. The descriptive statistics are demonstrated in Table 7 and detailed ANOVA results are presented in Table 8.

A significant interaction was observed between material and application (*p* = 0.010) and between application and time (*p* = 0.004). However, the interaction between material and time (*p* = 0.465) and the three-way interaction among material, application, and time (*p* = 0.990) were not statistically significant (Table 8).

Tukey post hoc analysis demonstrated that GrandioSO exhibited significantly higher surface roughness values than all other materials (*p* < 0.001). Grandio Blocs also showed significantly higher roughness than G-ænial Universal Injectable and TriniQ (*p* < 0.001), whereas no statistically significant difference was observed between G-ænial Universal Injectable and TriniQ (*p* = 0.471).

Regarding the application, the acid group showed significantly higher surface roughness values than the distilled water group (*p* = 0.019), while no statistically significant differences were observed between acid and Gaviscon liquid (*p* = 0.505) or between Gaviscon liquid and distilled water (*p* = 0.244).

For the time factor, measurements both after acid challenge and after application showed significantly higher surface roughness values compared with the baseline (*p* < 0.001). However, the difference between measurements after acid challenge and after application was not statistically significant (*p* = 0.263).

### 3.4. Scanning Electron Microscopy

At the initial stage (T_0_), all materials were observed to display greater homogeneity and uniformity on their surfaces, with minimal presence of microcracks and porosity and stable matrix–filler bonding. Following the primary exposure to HCl (T_1_), surface matting and initial micro-erosion were observed, while polishing and cutting marks began to diminish. In groups continuously exposed to HCl (T_2_), significant surface irregularities, cracks and pores, matrix erosion, filler detachment and exposure, and micro-crater formation were observed. In the group treated with antiacid medication (T_2_), a stable appearance with reduced new deep crater formation was observed, but the previous surface degradation had not been fully resolved.

When material-based differences were considered, although GrandioSO appeared relatively stable, nanoclusters and filler particles remained detectable in both the HCl- and antiacid-treated groups, indicating acid-induced deterioration of the matrix–filler interface. In contrast to the other materials, the G-aenial Injectable composite exhibited void- and bubble-like formations on the surface after the second acid challenge at T_2_. However, a more homogeneous and stable surface morphology was observed in the antiacid-treated group.

In the TriniQ 3D-printed resin group, a homogeneous matrix–filler structure was evident even at the early stages, and specimens exposed to antiacid medication demonstrated a smoother surface compared with those subjected to continued acid exposure.

**Figure 2:**
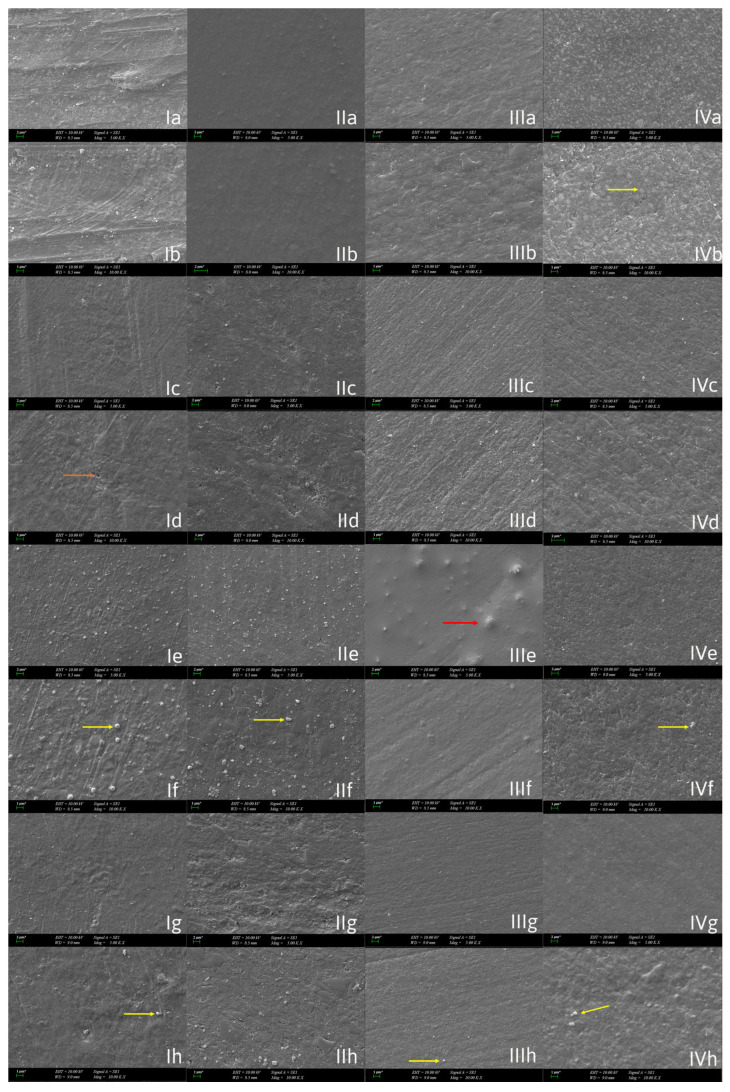
SEM images are provided at ×5000 magnification and ×10,000 magnification for the materials at T_0_, T_1_, and T_2_. As a column; (**I**) Grandioblocs, (**II**) GrandioSO, (**III**) G-aenial Universal Injectable, (**IV**) VarseoSmile TriniQ. As a line; (**a**,**b**) T_0_, (**c**,**d**) T_1_, (**e**,**f**) T_2_ (after HCl), (**g**,**h**) T_2_ (after antiacid medication). * Scale bar: represents the length indicated next to the bar in each image. Orange arrow: filler fall out, yellow arrow: filler, red arrow: bubble-like formation.

For Grandio Blocs, filler particles became apparent on the surface following acid exposure due to filler fallout. Nevertheless, in the antiacid-treated group, fewer exposed fillers were observed, which may be attributed to partial surface coverage compared with the continuously acid-treated specimens (Figure 2).

## 4. Discussion

Gastric acid exposure resulted in a significant increase in surface roughness and evident surface degradation in all tested resin-based materials; therefore, the first hypothesis was accepted. The second hypothesis was partially accepted, since gastric acid significantly reduced translucency and altered fluorescence values, whereas antiacid medication did not restore the surface and optical properties to baseline levels, despite limiting further deterioration compared with continued acid exposure. Since the surface and optical responses varied significantly among materials with different compositions and manufacturing techniques, indicating a clear material-dependent behavior under acidic conditions, the third hypothesis was accepted.

Studies assessing the effects of gastric acid on restorative dental materials have used a wide range of exposure protocols to simulate clinical conditions. However, there is no clear consensus regarding the equivalence between in vitro immersion times and clinical exposure durations [24]. Reported simulation periods vary substantially among studies due to differences in acid pH, temperature, exposure frequency, and the clinical conditions being modeled, such as gastroesophageal reflux or chronic vomiting [27]. For example, immersion times reported in the literature include 96 h (simulating over 10 years of clinical exposure) [28], 30 h (approximately 3 years) [23], and 18 h (equivalent to 8 years of vomiting in some studies) [29]. For shorter-term simulations, some studies suggest 45 min to 7.5 h as corresponding to 1 month of clinical exposure, 45 h to 6 months, and 91 h to 1 year [30]. As a result, identical immersion times may represent different clinical durations depending on the protocol. Nevertheless, prolonged gastric acid exposure consistently leads to greater material degradation, highlighting the cumulative erosive effect. In the present study, we specifically simulated GERD-related acid exposure (30 h-approximately 3 years) [23] to reflect long-term clinical conditions, and the samples were subjected to 37 °C for 6 days (144 h) as an extended erosive challenge.

Exposure to gastric acid has been shown to induce notable surface deterioration in resin-based restorative materials. In resin composites, this degradation has been associated with filler particle dislodgement and irregular dissolution of the resin matrix, as observed under SEM, accompanied by a marked increase in surface roughness values [5]. Consistent with these observations, reductions in surface microhardness following gastric acid immersion have also been reported, with SEM–EDS analyses revealing microstructural disruptions and elemental changes within the material surface [7]. İnci et al. observed distinct erosive surface patterns in pediatric restorative materials after gastric acid immersion, including surface porosities and matrix degradation, as revealed by SEM imaging [8]. Accordingly, surface roughness analysis and SEM imaging were performed to provide complementary quantitative and qualitative assessments of gastric acid–induced surface changes [5].

With respect to indirect restorative materials, gastric acid exposure has been reported to adversely affect surface topography and mechanical performance, although the extent of these changes appears to be material-dependent [11]. Such variability highlights the importance of microstructural composition in determining resistance to acidic environments, a finding that aligns with the material-specific responses observed in the present study. Systematic evidence further supports this variability, indicating that acidic solutions consistently increase surface roughness and reduce microhardness of indirect restorative materials, while the magnitude of these effects is influenced by both material composition and manufacturing technique [6]. This systematic evidence supports the observed variability among hybrid blocks and resin-based materials in the present study. In agreement with this evidence, the present study further demonstrated that the Grandio Blocs and the GrandioSO exhibited greater increases in surface roughness following acid exposure, which may be attributed to their heterogeneous microstructure and high filler loading, making the resin-rich regions more susceptible to acid-mediated degradation and filler exposure [6,17]. In hybrid CAD/CAM materials, the coexistence of resin and ceramic phases results in heterogeneous microstructures, which may create vulnerable regions at phase boundaries when subjected to chemical challenges [17]. Such structural heterogeneity offers a plausible explanation for the surface alterations observed in hybrid materials following acidic exposure in the current study. In contrast, the injectable composite and the 3D-printed resin showed comparatively lower surface roughness values both at baseline and after acid exposure, which may be related to their more homogeneous microstructural characteristics [14,18]. In the injectable composite, the uniform distribution of nano-sized fillers and effective filler–matrix coupling may have contributed to improved surface stability [15]. In contrast, in the 3D-printed material, controlled polymerization and post-curing processes associated with additive manufacturing may have promoted a more uniform matrix structure, thereby limiting acid-induced surface degradation [19,20]. In the present study, although gastric acid exposure caused a statistically significant increase in surface roughness in all materials, the G-ænial Injectable composite and the TriniQ 3D-printed resin consistently exhibited Ra values below the critical 0.2 µm threshold at all evaluation periods, a level that is considered clinically favorable due to its association with reduced bacterial adhesion, plaque accumulation, and risk of secondary caries [31].

Rodrigues et al. reported that acidic exposure negatively affects color and optical stability of dental ceramics, with surface roughness acting as a key mediator of optical degradation [9]. This relationship supports the concurrent evaluation of surface roughness and optical parameters in erosion-related studies. In addition, Rodrigues et al. noted that while optical properties such as color and translucency have been widely investigated following acidic exposure, fluorescence has rarely been included as an outcome parameter [9]. Therefore, in the present study, optical properties were evaluated by assessing both translucency and fluorescence to provide a more comprehensive characterization of acid-induced optical alterations.

Surface-induced optical changes have also been shown to extend beyond color-related parameters. Acid-induced surface degradation in dental ceramics has been associated with altered light transmission and scattering, leading to measurable changes in translucency and opacity [10]. The translucency changes observed in the present study may therefore be linked to acid-related surface morphological alterations rather than bulk material changes. However, despite acknowledging the broader impact of surface changes on optical behavior, previous studies did not address fluorescence characteristics under acidic conditions [10]. In the present study, increased surface roughness was associated with higher fluorescence alterations, revealing a relationship between surface characteristics and optical features. Acid-induced surface degradation may disrupt light transmission and promote irregular scattering, thereby masking the intrinsic fluorescence of resin-based materials [32,33].

For additively manufactured materials, evidence remains limited but suggests increased susceptibility to acidic environments. Gastric acid exposure has been shown to significantly increase surface roughness and color change in 3D-printed permanent resins, indicating their vulnerability to chemical degradation [20]. In addition, impaired bonding performance between 3D-printed acrylic materials and denture teeth following gastric acid exposure has been reported, suggesting degradation at the material interface [34]. Such chemical susceptibility may also contribute to surface and optical changes observed in 3D-printed resins.

Taken together, while previous investigations have predominantly focused on either mechanical performance or color-related outcomes, the present study offers a more comprehensive evaluation by simultaneously assessing surface roughness, surface morphology, translucency, and fluorescence across direct, CAD/CAM, and additively manufactured restorative materials under simulated GERD-related conditions. Alginate-based antiacids such as Gaviscon are designed not only to neutralize gastric acidity but also to form a viscous barrier upon contact with acid, thereby reducing acid contact with the surface [35]. In the present study, immersion in antiacid medication after the initial HCl challenge did not further increase surface roughness, and SEM observations suggested a more stable surface appearance with fewer newly formed deep craters compared with continued HCl exposure, which is consistent with a buffering/barrier effect rather than an additional erosive challenge. Importantly, previous in vitro evidence indicates that antiacid syrups can still induce material-dependent surface changes even when profilometric roughness does not increase [24]. In the study by Öcal and Dayı evaluating antiacid gastric syrups, exposure to Gaviscon Liquid produced significant reductions in giomer roughness over a 28-day regimen, while microhardness decreased significantly in both the microhybrid and giomer composites. Their SEM observations concurrently revealed increased surface irregularities and residues, including marked particle accumulation and irregularity on microhybrid surfaces after antiacid syrup and an increased number of pits/holes in giomer specimens. Together, these findings support the notion that alginate-antiacid formulations may attenuate acid-driven breakdown yet still modify surface integrity through residue formation and/or resin–filler interactions. To improve the generalizability and robustness of these findings, further studies with a broader range of restorative materials and antiacid formulations are needed, as the current evidence remains limited.

This study has certain limitations. As an in vitro investigation, the experimental conditions could not fully replicate the complex oral environment, including the buffering capacity of saliva and temperature fluctuations. The gastric acid exposure protocol was designed to simulate cumulative acidic challenges; however, individual variations in reflux frequency, duration, and severity in clinical settings may lead to different outcomes. The study also did not include an artificial saliva group as a positive control, which may have provided additional insight into the protective effects under more physiologically relevant conditions. SEM was used to evaluate surface morphology, providing primarily qualitative information, and future studies combining SEM with quantitative profilometry can achieve a more comprehensive and objective assessment of surface characteristics. PICN (Polymer-Infiltrated Ceramic Network) materials were not evaluated, and future research including them could provide a more comprehensive comparison. In addition, only one shade and a limited number of resin-based materials were evaluated, which may restrict the generalizability of the findings. Therefore, further clinical studies incorporating a wider range of materials and patient-related variables are needed to better understand these effects.

## 5. Conclusions

Within the limitations of this in vitro study, gastric acid exposure was shown to negatively influence the clinical performance of resin-based restorative materials by affecting both surface integrity and optical stability. Antiacid medication reduced further surface deterioration. From a clinical perspective, G-aenial Injectable and TriniQ showed better performance, highlighting the importance of long-term monitoring of restorations in patients with gastroesophageal reflux disease, as repeated acidic challenges may compromise esthetic outcomes and restoration durability over time. Under acid challenge conditions, restorations may benefit from more frequent evaluations, with periodic polishing or surface maintenance helping to preserve material integrity and reduce acid-induced damage.

## Figures and Tables

**Table 1:** Composition of the resin-based materials used.

Material (Shade)	Manufacturer	Type	Content
VarseoSmile TriniQ (A2)	BEGO GmbH, Germany	CAD/CAM 3D-printed resin composite	4,4′-isopropylidenediphenol ethoxylated, 2-methylprop-2-enoic acid, benzeneacetic acid, α-oxo-methyl ester, photoinitiator: TPO. Filler (wt):NR
Grandio Blocs HT (A2)	VOCO, Cuxhaven, Germany	Nano-ceramic hybrid CAD/CAM indirect resin composite	UDMA + DMA (14 wt%), nanohybrid fillers: nanosilica + barium glass. Filler (wt): 86%
GrandioSO (A2)	VOCO, Cuxhaven, Germany	Nano-hybrid resin composite	Bis-GMA, Bis-EMA, TEGDMA, Glass-ceramic filler (~1 µm) + SiO_2_ nanoparticles (20–40 nm). Filler (wt): 89%
G-ænial Universal Injectable (A2)	GC Corp., Japan	Nano-hybrid resin composite	UDMA, Bis-MEPP, TEGDMA, pigments, photoinitiator, SiO_2_ + strontium glass (10–200 nm). Filler (wt): 69%

Abbreviations: CAD/CAM, computer-aided design/computer-aided manufacturing; HT, high translucency; wt%, weight percent; NR, not reported; UDMA, urethane dimethacrylate; DMA, dimethacrylate; Bis-GMA, bisphenol A-glycidyl methacrylate; Bis-EMA, ethoxylated bisphenol A dimethacrylate; TEGDMA, triethylene glycol dimethacrylate; Bis-MEPP, 2,2-bis(4-methacryloxypolyethoxyphenyl)propane; TPO, diphenyl(2,4,6-trimethylbenzoyl)phosphine oxide.

**Table 2:** Composition of solutions used in the study.

Solutions	Manufacturer	pH	Content
Gaviscon Liquid	Reckitt Benckiser, Slough, UK	9.0	Active Ingredient: Calcium Carbonate (80 mg), Sodium Bicarbonate (133.5 mg) Other Components: Methyl Paraben 20 mg, Propyl Paraben 3 mg
Distilled Water		7.4	
Gastric Acid		1.2	

**Table 3:** Descriptive statistics of the translucency of materials at initial (T_0_), after acid challenge (T_1_), and after different applications (T_2_).

Materials	Applications	T_0_	T_1_	T_2_	Total
Grandio Blocs	Acid	18.22 ± 1.23	16.39 ± 1.07	16.23 ± 0.9	16.93 ± 1.74 ^a^
	Gaviscon Liquid	18.17 ± 2.05	16.32 ± 1.75	14.65 ± 1.6	
	Distilled water	18.69 ± 1.19	16.79 ± 0.82	16.88 ± 0.84	
GrandioSO	Acid	24.62 ± 2.11	18.22 ± 1.33	18.02 ± 1.15	19.53 ± 3.71 ^b^
	Gaviscon Liquid	23.24 ± 1.7	16.47 ± 1.17	14.82 ± 1.27	
	Distilled water	24.35 ± 1.33	17.97 ± 1.76	18.03 ± 1.77	
G-ænial Universal Injectable	Acid	19.56 ± 1.96	17.76 ± 0.97	17.06 ± 0.89	17.88 ± 1.76 ^c^
	Gaviscon Liquid	18.88 ± 1.21	18.63 ± 2.56	17.01 ± 1.07	
	Distilled water	18.7 ± 2.09	16.65 ± 0.64	16.68 ± 0.93	
TriniQ	Acid	20.08 ± 1.63	17.44 ± 0.77	16.79 ± 1.34	18.69 ± 2.07 ^d^
	Gaviscon Liquid	21.76 ± 0.97	18.11 ± 1.58	16.99 ± 1.34	
	Distilled water	20.66 ± 0.92	18.25 ± 1.53	18.14 ± 1.47	
Total		20.58 ± 2.73 ^A^	17.42 ± 1.58 ^B^	16.77 ± 1.6 ^C^	
Total	Acid	18.37 ± 2.54 ^xy^			
	Gaviscon Liquid	17.92 ± 2.87 ^x^			
	Distilled water	18.48 ± 2.45 ^y^			

There is no statistically significant difference between the same letters.

**Table 4:** Comparison of translucency values according to material type, solution and measurement time.

Source	F	*p*	Partial Eta Squared
Corrected Model	22.989	<0.001	0.736
Material	49.020	<0.001	0.338
Application	4.643	0.010	0.031
Measurement time	218.920	<0.001	0.603
Material*Application	7.862	<0.001	0.141
Material*time	23.435	<0.001	0.328
Application*time	3.510	0.008	0.046
Material*Application*time	0.716	0.736	0.029

F: Analysis of Variance test statistic; R^2^ = 0.736 (Adjusted R^2^ = 0.704). “*” sign was used for interactions.

**Table 5:** Descriptive statistics of the fluorescence difference of materials between initial and after acid challenge (T_0_–T_1_), and between initial and after different applications (T_0_–T_2_).

Materials	Applications	T_0_–T_1_	T_0_–T_2_	Total
Grandio Blocs	Acid	2.53 ± 0.46	3.4 ± 0.92	2.56 ± 0.76 ^a^
	Gaviscon Liquid	2.12 ± 0.56	2.96 ± 0.85	
	Distilled water	2.14 ± 0.34	2.2 ± 0.37	
GrandioSO	Acid	1.85 ± 0.13	2.36 ± 0.58	2.86 ± 1 ^a^
	Gaviscon Liquid	3.23 ± 0.94	3.47 ± 0.78	
	Distilled water	3.07 ± 1.07	3.16 ± 1.27	
G-ænial Universal Injectable	Acid	4.2 ± 1.02	4.29 ± 0.98	4.19 ± 1 ^b^
	Gaviscon Liquid	4.05 ± 1.19	4.63 ± 1.31	
	Distilled water	3.88 ± 0.68	4.11 ± 0.79	
TriniQ	Acid	4.63 ± 1.02	5.38 ± 1.3	4.46 ± 1.14 ^b^
	Gaviscon Liquid	3.79 ± 1.07	4.17 ± 1.28	
	Distilled water	4.35 ± 0.67	4.43 ± 1	
Total		3.32 ± 1.22	3.71 ± 1.31	
Total	Acid	3.58 ± 1.44		
	Gaviscon Liquid	3.55 ± 1.23		
	Distilled water	3.42 ± 1.17		

There is no statistically significant difference between the same letters.

**Table 6:** Comparison of fluorescence difference according to material type, solution and measurement time.

Source	F	*p*	Partial Eta Squared
Corrected Model	9.888	<0.001	0.542
Material	57.810	<0.001	0.475
Application	0.654	0.521	0.007
Measurement time	9.922	0.002	0.049
Material*Application	6.196	<0.001	0.162
Material*time	0.326	0.806	0.005
Application*time	1.261	0.286	0.013
Material*Application*time	0.349	0.910	0.011

F: Analysis of Variance test statistic; R^2^ = 0.542 (Adjusted R^2^ = 0.487). “*” sign was used for interactions.

**Table 7:** Descriptive statistics of the surface roughness values (Ra) in μm of materials at initial (T_0_), after acid challenge (T_1_), and after different applications (T_2_).

Materials	Applications	T_0_	T_1_	T_2_	Total
Grandio Blocs	Acid	0.17 ± 0.04	0.2 ± 0.04	0.21 ± 0.05	0.2 ± 0.03 ^a^
	Gaviscon Liquid	0.2 ± 0.01	0.21 ± 0.01	0.21 ± 0.02	
	Distilled water	0.19 ± 0.03	0.21 ± 0.02	0.2 ± 0.03	
GrandioSO	Acid	0.22 ± 0.04	0.25 ± 0.05	0.28 ± 0.05	0.24 ± 0.04 ^b^
	Gaviscon Liquid	0.22 ± 0.01	0.25 ± 0.01	0.25 ± 0.01	
	Distilled water	0.22 ± 0.03	0.24 ± 0.02	0.23 ± 0.02	
G-ænial Universal Injectable	Acid	0.09 ± 0.01	0.1 ± 0.01	0.11 ± 0.01	0.09 ± 0.02 ^c^
	Gaviscon Liquid	0.08 ± 0.01	0.09 ± 0.01	0.09 ± 0.01	
	Distilled water	0.08 ± 0.02	0.09 ± 0.02	0.1 ± 0.02	
TriniQ	Acid	0.09 ± 0.01	0.1 ± 0.02	0.13 ± 0.02	0.1 ± 0.02 ^c^
	Gaviscon Liquid	0.09 ± 0.02	0.1 ± 0.02	0.1 ± 0.03	
	Distilled water	0.09 ± 0.02	0.1 ± 0.02	0.1 ± 0.02	
Total		0.14 ± 0.06 ^A^	0.16 ± 0.07 ^B^	0.17 ± 0.07 ^B^	
Total	Acid	0.16 ± 0.07 ^x^			
	Gaviscon Liquid	0.16 ± 0.07 ^xy^			
	Distilled water	0.15 ± 0.07 ^y^			

There is no statistically significant difference between the same letters.

**Table 8:** Comparison of surface roughness values (Ra) according to material type, solution and measurement time.

Source	F	*p*	Partial Eta Squared
Corrected Model	64.417	<0.001	0.887
Material	717.163	<0.001	0.882
Application	3.752	0.025	0.025
Measurement time	26.675	<0.001	0.156
Material*Application	2.843	0.010	0.056
Material*time	0.943	0.465	0.019
Application*time	4.007	0.004	0.053
Material*Application*time	0.292	0.990	0.012

F: Analysis of Variance test statistic; R^2^ = 0.887 (Adjusted R^2^ = 0.873). “*” sign was used for interactions.

## Data Availability

The raw data supporting the conclusions of this article will be made available by the authors on request.

## Abbreviations

The following abbreviations are used in this manuscript:

GERD	Gastroesophageal reflux disease
CAD/CAM	Computer-aided design/computer-aided manufacturing
HT	High translucency
wt%	Weight percent
NR	Not reported
UDMA	Urethane dimethacrylate
Bis-GMA	Bisphenol A-glycidyl methacrylate
Bis-EMA,	Ethoxylated bisphenol A dimethacrylate
TEGDMA	Triethylene glycol dimethacrylate
Bis-MEPP	2,2-bis(4-methacryloxypolyethoxyphenyl)propane
TPO	Diphenyl(2,4,6-trimethylbenzoyl)phosphine oxide
PICN	(Polymer-Infiltrated Ceramic Network)
